# Bad Teeth vs. Good Health

**Published:** 1910-11-15

**Authors:** J. J. McCarthy


### BAD TEETH VS. GOOD HEALTH.

BY J. J. McCARTHY, M.D.

There are in this country eight factories devoted to the manufacture of artificial teeth. Last year the manufacturers sold over 60,000,000 of these teeth and this year they expect to sell between 78,000,000 and 80,000,000; and every one of these teeth goes to replace a natural tooth, which, if given proper care and attention, should last out one's lifetime. Unclean mouths and teeth are responsible for these conditions, for it is a fact fully established that less than 8 per cent. of the American people use a tooth brush or make any effort to keep their teeth and mouths clean. In order to have good health, we must have sound teeth, yet we are permitting our teeth to decay at a pace that is alarming, which, if unchecked, will lead to a nation of broken-down, dyspeptic men and women.

The sixth year molar is the most important of all teeth. It is often lost because parents frequently think that it is one of the temporary set. This error is due to the fact that it is cut while most of the first set of teeth are in the mouth. When this tooth is lost, Nature makes an attempt to close the space, with the result that the entire articulation is destroyed. The space thus created between the teeth is difficult to keep clean, due to the food particles being forced into these spaces. Its loss is also one of the chief causes of irregularities of the other erupting teeth. It is the belief of the entire dental profession that the early loss of this tooth is responsible for more misplaced teeth and ill-shaped jaws than any other condition of the mouth. Dr. Woodbury, the noted neurologist of Boston, has called these molar teeth the "working tools of mastication." He says: "their work begins at once and continues throughout life. Upon them rest full growth and development. Upon them depends good health during life." When this tooth appears, about the sixth year, it is frequently found decayed within the year following. It is at this time that children acquire a fondness for sweets of every description, and having not learned the habit of brushing their teeth and properly cleaning them, this tooth in particular becomes affected and rapidly decays. With the defect in articulation caused by the loss of this tooth, the proper chewing of food is not possible, with the result that children and adults as well become habitual "food bolters." It should be the particular duty of every mother to become familiar with the location of this important tooth. Beginning at the center in front and counting backward on either side, above and below, it is the sixth tooth cut. The mother should carefully watch for any defects found in this tooth and if cavities are located they should be repaired at once in order that the usefulness of the tooth can be saved.

The great American habit, the "bolting of food," is one of the most serious conditions of our modern life. Dr. Osler has said that the American nation could be divided into two classes, bolters and chewers, with the bolters leading by a large majority. Dr. D. H. Sexton, of Shelbyville, Ind., at a recent meeting of the Indiana Dental Association, delivered an interesting address in which he deplored this habit, and advised that a national movement should be organized to be known as the "Chewing Movement." He said: "The education of the average man, woman and child has been sadly neglected. They have been taught to eat, but have not been taught to use their teeth. When we bolt our food, we ignore one of the most important ferments, ptyalin, in our saliva, that has much to do in the process of digestion. But the American habit is to spit, and Americans are the greatest spitters of the world. Between meals they will spit out the invaluable saliva; then when they eat they wash down every unchewed bolus of food with copious draughts of water, coffee, or in summer iced tea. What a foolish, disgusting habit it is and more than foolish, more than disgusting, it is killing in its hurtfulness. An habitual spitter at middle age will have the broken down digestive apparatus of an old man at seventy-five. Men who bolt their food, who put their saliva out of business, are drug shop chasers and slow suicides."

Dr. Henry C. Ferris, recording secretary of the New York State Dental Society, recently presented an illuminating report showing the effects of the bolting of food. Dr. Ferris addressed a letter to one hundred and fifty of the prominent medical men of this country in which he asked them if they considered imperfect chewing and salivating of food an etiological factor in diseases of the stomach and intestines, and, if so, what pathological conditions resulted from such neglect? Out of the hundred and fifty replies that Dr. Ferris received 98 per cent. of these physicians said that chewing of food was an important factor toward good health and that the bolting of food frequently caused cancer, catarrh of the stomach and gastric ulcers. If food is not thoroughly chewed and is permitted to reach the stomach in large lumps or masses, there is no question that it must injure the soft lining of that organ, producing many of the cases of ulcer and catarrh that need careful and consistent medical attention.

It has been stated that fully 75 per cent. of the people of this country bolt their food. This habit is usually acquired during the early years of childhood and carried on during one's whole life. In many of the homes, the early morning hours are given to preparing the children for school. Very frequently they are permitted to sleep late and in the hurry and bustle to get them to school on time, the breakfast is bolted. These same conditions of hurried meals apply to the lunch hour and supper time. From day to day this is permitted, until the habit is firmly established, carried up to manhood, and then down through old age. We have a lesson every day of the bolting of food. Walk into the quick-lunch room of the cities and see these "hustlers" at work. Look down the long row of tables, see the rapid movement of the diners, and you will liken it to a quick-eating contest, for which prizes are offered to the fellow that gets through first. Many of these lunch rooms advertise how quickly you may be filled from their larder and take a pride in the number that can be served in a given time. No doubt much of their trade comes from people who want their eating over in a hurry. The average business man almost begrudges the time given to eating; it is rarely a pleasure with him, especially the lunch hour repast, and he goes at it in a vigorous, may I call it a pugilistic, way, and fights the food to a finish. As he walks out he seems to say "Well, that thing is over." When his stomach, as all stomachs will when given such bad treatment, rebels and he becomes a chronic sufferer from indigestion, he wonders how it all happened. The doctor knows, but the advice many times is too late, and, if given, is often forgotten.

One authority has said, "Americans are discarding the use of their teeth; they have adopted a new fad; they are becoming a race of bolters. In the next three thousand years the people of this country will be toothless. As evolution progresses, the teeth will disappear and the man or woman with teeth will be a curiosity. Our ideas of beauty will have changed and as everyone will have no teeth we won't mind the change in facial make-up. Beauty changes with evolution, so we will have accustomed ourselves to an era of toothless men and women."

It should be the duty of all parents to make their children eat slowly and chew their food properly. Nature has placed these teeth in our mouth for a purpose, a very valuable purpose, too, and if we neglect to properly use them, we are inviting conditions that seriously threaten our health and general welfare.

The school inspectors of Cleveland, Ohio, examined 33,000 children last year and discovered 77 per cent. of them to have defective teeth. In four schools 2,672 children were examined and less than 1 per cent. had perfect teeth. In these four schools alone, 15061 teeth were found that needed immediate attention if they were to be saved. In Richmond, Va., only 65 out of 1,173 children examined had no defective teeth. In one school in Detroit out of 305 children 34 per cent. had diseased teeth, with 1,304 tooth cavities. In the public schools of Boston the visiting nurses found that 75 per cent. of the school children had decayed teeth, and in Atlanta the school inspectors found that 60 per cent. of the children needed dental attention. In Chicago the examination has not progressed far enough to give definite statistics, but those in charge of the work say that the returns will be fully as bad as the conditions reported by the nurses in Boston. As each city takes up the work of inspecting the children's teeth, additional facts are brought to light, showing that defective and diseased teeth exceed all other physical defects of the school children of this country. It has been estimated that there are over 9,000,000 children in the United States who have defective and diseased teeth and 5,000,000 with enlarged glands and 7,000,000 with defective breathing due to adenoid growths in the nose. These estimates are strictly conservative and show what condition exists among the children of this country. Dr. O. W. White, a well-known orthodontist, of Detroit, says that 80 per cent. of children suffer from mal-occlusion of the teeth or undeveloped mouths and about 20 per cent. are habitual mouth breathers.

The same conditions prevail abroad. In England it was found that 80 per cent. of the children in the industrial schools are suffering from decayed teeth and 75 per cent. of the children in the British Isles have unclean mouths.

In Germany, out of 20,000 children examined, ranging between the ages of six and sixteen years, 95 per cent had dental caries.

In Frankfurt, of 1,020 children examined there were found 28,673 defective teeth, and fully 50 per cent. of these children were suffering in general health as a result of these conditions.

It is a fact that 90 per cent. of all the children in Germany, England and in the United States are beginning life seriously handicapped with defective teeth.

Dental caries frequently originates during pregnancy and should receive immediate attention. There is an old and true saying, "a tooth for every child," but this can be avoided if the prospective mother will follow the instructions of her physician and her dentist Dr. A. F. Strange, president of the Central Illinois Dental Society, in explaining these conditions, says: "Decay of the teeth is more usual in women because of indoor life and lack of exercise in the open air. This is increased greatly during the severe strain on the system, especially during the physiological process of reproduction. The importance of caring for the teeth during this period cannot be too strongly emphasized. We are often asked: Is it advisable for a woman to have her teeth given any attention during this period? Most emphatically, yes. It is just as safe and brings great relief to the expectant mother, and at the same time prevents the transmission of irritability and nervousness to the unborn child. In this modern age of civilization, the prospective mothers should know that as they take care of their own health, so will be the health of the unborn babe."

The rapid decay of teeth as seen in most women during his critical period is not due to the defficiency of lime salts in the blood as was at first generally supposed, but to acid eructations, vomiting and regurgitations from a disordered stomach. It is well known that during this period the mouth is bathed in an acid saliva and tooth dectruction is rapid.

It is not uncommon to find mothers following childbirth with from eight to twelve cavities in their teeth. In many instances teeth that have been filled lose their filling following this period, and the dentist is frequently accused of having used inferior material or of being negligent. This is an injustice to the dentist and is due to the ignorance of the mother as to proper attention in the matter of food and diet during the reproductive period. The prospective mother should eat plenty of food rich in lime salts and frequently take either hyposulphite of lime or syrup of wheat phosphates, both excellent bone forming tonics. In order to overcome the excessive acidity of the mouth, she should rinse her mouth several times a day with milk of magnesia. This is an alkaline preparation which neutralizes all acids in the mouth, coating the teeth with an alkaline film that protects the tooth surface for hours, thus preventing decay. —*Pearsons Magazine.*

